# CRISPR/Cas9 in Cancer Immunotherapy: Animal Models and Human Clinical Trials

**DOI:** 10.3390/genes11080921

**Published:** 2020-08-11

**Authors:** Khalil Khalaf, Krzysztof Janowicz, Marta Dyszkiewicz-Konwińska, Greg Hutchings, Claudia Dompe, Lisa Moncrieff, Maurycy Jankowski, Marta Machnik, Urszula Oleksiewicz, Ievgeniia Kocherova, Jim Petitte, Paul Mozdziak, Jamil A. Shibli, Dariusz Iżycki, Małgorzata Józkowiak, Hanna Piotrowska-Kempisty, Mariusz T. Skowroński, Paweł Antosik, Bartosz Kempisty

**Affiliations:** 1 Department of Anatomy, Poznan University of Medical Sciences, 60-781 Poznań, Poland; khalilkhalaf0216@gmail.com (K.K.); krzysztof.janowicz.16@abdn.ac.uk (K.J.); m.dyszkiewicz@ump.edu.pl (M.D.-K.); g.hutchings.16@abdn.ac.uk (G.H.); mjankowski@ump.edu.pl (M.J.); kocherova.evgenia@gmail.com (I.K.); 2 The School of Medicine, Medical Sciences and Nutrition, University of Aberdeen, Aberdeen AB25 2ZD, UK; claudia.dompe.16@abdn.ac.uk (C.D.); l.moncrieff.16@abdn.ac.uk (L.M.); 3 Department of Biomaterials and Experimental Dentistry, Poznan University of Medical Sciences, 60-812 Poznań, Poland; 4 Department of Histology and Embryology, Poznan University of Medical Sciences, 60-781 Poznań, Poland; 5 Department of Cancer Immunology, Poznan University of Medical Sciences, 60-408 Poznan, Poland; marta1machnik@gmail.com (M.M.); u.oleksiewicz@gmail.com (U.O.); dmizy@ump.edu.pl (D.I.); 6 Department of Cancer Diagnostics and Immunology, Greater Poland Cancer Centre, 61-866 Poznan, Poland; 7 Prestage Department of Poultry Science, North Carolina State University, Raleigh, NC 27695, USA; jnppo@ncsu.edu; 8 Physiology Graduate Program, North Carolina State University, Raleigh, NC 27695, USA; pemozdzi@ncsu.edu; 9 Department of Periodontology and Oral Implantology, Dental Research Division, University of Guarulhos, Guarulhos 07023-070, Brazil; jashibli@yahoo.com; 10 Department of Toxicology, Poznan University of Medical Sciences, 61-631 Poznań, Poland; malgorzata.jozkowiak@gmail.com (M.J.); hpiotrow@ump.edu.pl (H.P.-K.); 11 Department of Basic and Preclinical Sciences, Institute of Veterinary Medicine, Nicolaus Copernicus University in Torun, 87-100 Toruń, Poland; skowron@umk.pl; 12 Department of Veterinary Surgery, Nicolaus Copernicus University in Torun, 87-100 Toruń, Poland; pantosik@umk.pl; 13 Department of Obstetrics and Gynecology, University Hospital and Masaryk University, 601 77 Brno, Czech Republic

**Keywords:** genome editing, cancer, animal models, experimental oncology

## Abstract

Even though chemotherapy and immunotherapy emerged to limit continual and unregulated proliferation of cancer cells, currently available therapeutic agents are associated with high toxicity levels and low success rates. Additionally, ongoing multi-targeted therapies are limited only for few carcinogenesis pathways, due to continually emerging and evolving mutations of proto-oncogenes and tumor-suppressive genes. CRISPR/Cas9, as a specific gene-editing tool, is used to correct causative mutations with minimal toxicity, but is also employed as an adjuvant to immunotherapy to achieve a more robust immunological response. Some of the most critical limitations of the CRISPR/Cas9 technology include off-target mutations, resulting in nonspecific restrictions of DNA upstream of the Protospacer Adjacent Motifs (PAM), ethical agreements, and the lack of a scientific consensus aiming at risk evaluation. Currently, CRISPR/Cas9 is tested on animal models to enhance genome editing specificity and induce a stronger anti-tumor response. Moreover, ongoing clinical trials use the CRISPR/Cas9 system in immune cells to modify genomes in a target-specific manner. Recently, error-free in vitro systems have been engineered to overcome limitations of this gene-editing system. The aim of the article is to present the knowledge concerning the use of CRISPR Cas9 technique in targeting treatment-resistant cancers. Additionally, the use of CRISPR/Cas9 is aided as an emerging supplementation of immunotherapy, currently used in experimental oncology. Demonstrating further, applications and advances of the CRISPR/Cas9 technique are presented in animal models and human clinical trials. Concluding, an overview of the limitations of the gene-editing tool is proffered.

## 1. Introduction

Currently, cancer represents the second leading cause of deaths worldwide after cardiovascular diseases [1]. Therefore, the ongoing global burden of malignancies remains a large concern in medicine and oncology, demanding new treatment strategies [2]. However, despite the large advancements in cancer surgery, physical interventions have been demonstrated to elevate risks of metastatic recurrences by increasing the spread of cancer cells [3], as well as elevating pro-inflammatory mediators, angiogenic factors [4], or cancer dependent adhesion molecules [5]. Furthermore, the effectiveness of non-surgical cancer therapies depends on different factors, which include the type, severity of cancer, its metastatic potential, immunity status, as well as the age and physiological conditions of patients. Cancer immunity is an especially prominent factor influencing the effectiveness of the available treatment methods, including such important processes as the development of multi-drug resistance (MDR) mechanisms, immunity arrest, as well as formation of tumor microenvironment [6]. Therefore, even though multiple treatments are currently used to increase the specific for tumor control, genome editing techniques technology recently arose to be a viable alternative to other, more invasive methods including surgeries, chemotherapies, and immunotherapies [7]. It could be argued that CRISPR/Cas9, despite several limitations, is an especially promising representant of these new techniques, possibly allowing for precise editing of specific gene sequences [8]. Hence, this article presents an overview of the CRISPR/Cas9 method and its possible application in cancer treatment. Additionally, selected current animal model studies and clinical trials associated with the use of this methods are also presented.

## 2. Overview of the CRISPR/Cas9 Mechanism

**Figure 1:**
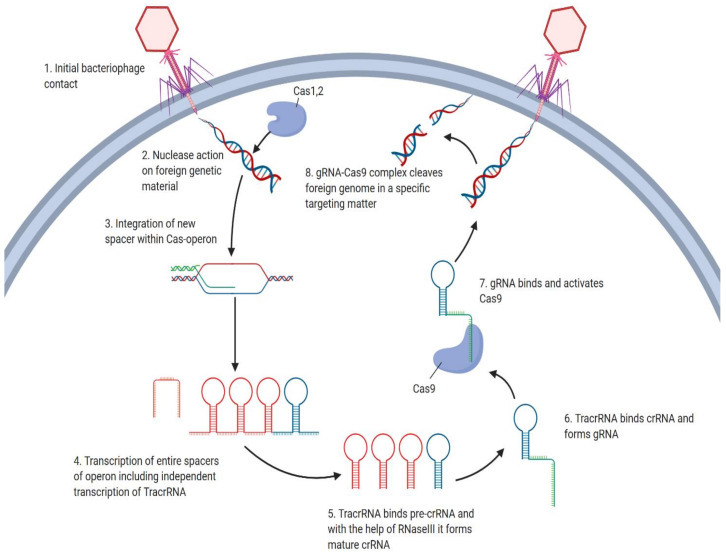
Once the invading genetic material from the bacteriophage enters the cell (1), CRISPR associated proteins 1 and 2 (Cas 1,2) will degrade the material (2), which will then be integrated as a spacer within the Cas-operon (3). Transcription of the entire spacer within the operon will take place, and TracrRNA will be transcribed independently (4). TracrRNA binds to pre-crRNA, and with the help of RNaseIII, cleavage will form individual and mature crRNA (5). The formation of gRNA occurs once tracrRNA binds to crRNA (6). gRNA binds Cas9 (7), redirecting it to the subsequent bacteriophage genetic insertion resulting in the destruction of foreign material (8). Terminologies: Cas9: CRISPR associated protein 9; gRNA: guide RNA (guides and activates insertion/deletion of Cas9 thus is used for activation and target specificity); tracrRNA: trans-activating CRISPR RNA (forms a complex with gRNA and acts as a homing device for directing Cas9 to invade foreign genomic material); crRNA: CRISPR RNA; PAM: Proto-spacer Adjacent Motif (a component of the virus or plasmid that plays an important role in target DNA selection and degradation); anHNH: nuclease system component of Cas9 that cleaves target DNA; RuvC-like: nuclease system component of Cas9 that cleaves the non-target DNA; RNAi: RNA interference (RNA molecule that inhibits gene expression/translation by neutralizing target mRNA).

**Figure 2:**
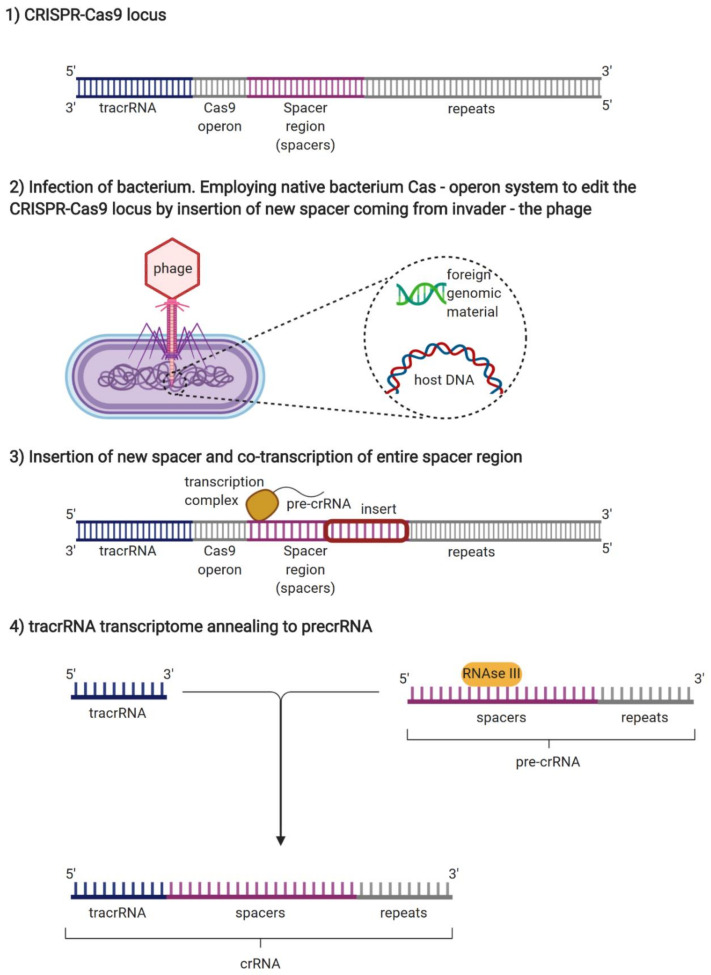

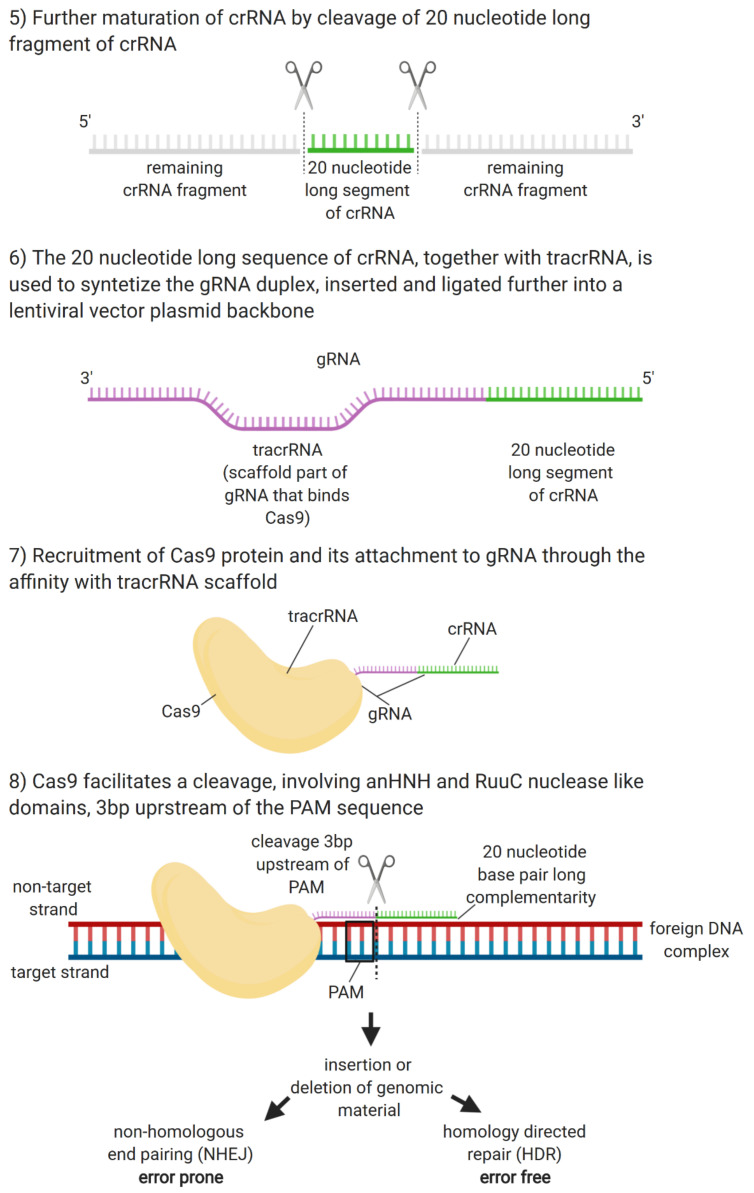
Overview of the CRISPR/Cas9 mechanism of action. The CRISPR method is based on the natural system used by bacteria to protect themselves from viral infections. When the bacteria detect the presence of viral DNA, they produce two short RNA sequences, one of which matches the sequence of the invading virus. These two RNAs form a complex with a protein called Cas9. Analogically, scientists firstly identify the gene causing a health problem, followed by the design of guide gRNA that recognizes that particular stretch of nucleotides. gRNA is attached to Cas9 nuclease and complex cuts DNA. At that point, researchers then edit an existing genome by modifying the leading strand or inserting foreign DNA sequence.

Discovery of a prokaryotic immune defense mechanism known as Clustered Regularly Interspaced Short Palindromic Repeats (CRISPR) created new possibilities for specifically targeting genetic diseases, particularly cancer, on a nucleotide level [8]. CRISPR/Cas9 systems are based on phage strategies for injection of foreign nucleic acids to maintain prokaryotic adaptive immunity. The CRISPR/Cas9 locus is associated by Cas9 operon, consisting of repeats interspersed by non-repetitive sequences called spacers (Figure 1), as well as tracrRNA, a unique noncoding RNA homologous to a stretch of nucleotides called repeat sequences, located upstream of the Cas9 operon [9]. Once the phage infects the bacterium, a foreign DNA, specifically spacer sequence, gets added to the host cell genome [10]. After spacer insertion, CRISPR array associated Cas9 operon facilitates the co-transcription of the entire spacer region resulting in the formation of an immature segment called pre-crRNA, consisting of newly added spacer and host DNA [11]. In other words, immature crRNA consists of repeats and spacers of both host and foreign origins [12]. Following co-processing of tracrRNA and pre-crRNA by RNase III, tracrRNA anneals to pre-crRNA, forming a mature segment called crRNA. Further maturation of crRNA to gRNA is achieved by cleavage of the crRNA segment to 20 nucleotides long sequence through the action of Cas1 and Cas2 nucleases [13]. CRISPR/Cas9 system requires Cas9 endonuclease to bind guide RNA (gRNA), forming a Cas9/gRNA complex. Thanks to guide RNA, the complex is directed for site-specific cleavage of the target DNA (Figure 1) [14]. PAM sequences in foreign DNA are localized by gRNA bound Cas9, complementary to each target sequence [15]. Double strand breaks in DNA occur three base pairs upstream from the PAM sequence via the action of two nuclease domains, the HNH domain and the RuvC-like nuclease [16]. Endonuclease Cas9 is inactive unless bound by gRNA, to ensure target specificity [17]. The resulting double strand breaks can be repaired by error-prone non-homologous end joining (NHEJ), allowing either insertion or deletion of specific sequence or error-free homologous directed repair (HDR) (Figure 2) [18].

## 3. Applications and Advances of the CRISPR/Cas9 Technique in Animal Cancer Model and Human Clinical Trials

CRISPR/Cas9 enables genome modification, potentially allowing for target correction of mutations. Furthermore, it can be used to generate and recreate mutations in vivo and consequently observe the development and progress of tumorigenesis in animal models [19]. This is advantageous, as due to the lack of data concerning embryonic stem cells and the efficiency of homologous recombination, large animals, including dogs and pigs, were only recently introduced to the array of gene-modified animals used for biological and biomedical research [20]. Moreover, there is a large potential in recreating human disease in animal models, as well as in vitro modeling of genetic variants in CRISPR/Cas9-edited cell lines, possibly allowing to develop the fields of gene therapy and regenerative medicine [21]. The establishment of cancer toolboxes aids the efficiency of gene therapies by allowing to control the expression of tumor suppressor genes, oncogenes, as well as access conditional transgenic approaches [22].

Somatic genome engineering with CRISPR/Cas9 and CRISPR/Cas9-based effectors, as well as genetic screens, complement therapeutic applications of gene and immune therapies in cancer biology [23]. As an example, even though genomic approaches allow to identify its low and moderate penetrance variants, in vivo modeling of recurrent ERCC3 truncating mutation, which contributes to moderate risk of breast cancer in *ERCC3*-deficient cells, allowed to observe effects of rescuing *ERCC3* repair-deficient phenotype [24]. This gene-editing system also allows for in vivo prediction of biomarkers of sensitivity to small molecules, e.g., the identification of *SLFN11*, sensitive to PARP inhibitors, as a predictive biomarker for small cell lung cancer [25]. Investigation of mutations in the second and third generation of EGFR-tyrosine kinase inhibitors in EGFR-mutant non-small cell lung cancer, led to the identification of causative mutation and consequently overcoming targeted resistance [26]. In other studies, genetically engineered mice used to study causative mutations in small cell lung carcinoma (SCLC) were transfected with adeno-associated viral vectors containing gRNAs specifically designed to target the SCLC predisposing mutations in tumor suppressors TP53 and RB1 [19]. Animal model studies demonstrated disease features similar to human SCLC, sharing characteristics such as the location of metastatic spread and histopathologic properties. As a result, mice genes downregulated by targeted mutations in TP53 and RB1 of SCLC were used as potential therapeutic targets in humans. Furthermore, gRNAs were specifically designed to target other genes, including p107 and p130, to understand their connection with the disease and pathogenicity. Following six months from tumor initiation, bioluminescence imaging, performed to track tumor development, delivered high luciferase activity in both gRNA-107 and gRNA-130 infected cells, proving their involvement in early metastasis of SCLC. Therefore, CRISPR proved efficient in recreating disease in animal models in addition to the identification of mutations in newly discovered genes responsible for SCLC metastasis [27].

Moreover, genome-scale CRISPR/Cas9 knockout (GeCKO) screens enabled the induction of mutations and identification of the cooperative function of candidate genes associated with *p53* and *KRAS* oncogenes in Kras^G12D^ immortalized mouse embryonic fibroblasts, aiding understanding of primary sarcomas in mice [28]. The human GeCKO library was also used to screen out six candidate genes believed to confer cisplatin resistance in ovarian cancer cell lines, leading to the identification of only two, *SULF1* and ZNF587B, the loss of which increased ciplatin resistance [29]. In turn, in yet another sarcoma study, complete knockout of ORF57 was achieved in iSLK/Bac16 and HEK293/Bac36 cells, simultaneously expressing one Cas9 protein and two guide RNAs associated by several rounds of single-cell cloning and cell selection from a single vector [30]. In a further trial, researchers investigated the role of the *FRK* oncogene in the H1299 lung cancer cell line, demonstrating that knockout of *FRK* suppressed epithelial to mesenchymal transition cell colony formation and proliferation of cancer cells [31]. In turn, in a study concerning canine cancer research, knockout of one of the main tumor suppressor genes, *TP53,* was rescued by administration of TP53 gRNA, resulting in in vivo and in vitro non-cancerous phenotypes [32]. The study further explored the potential *TP53*KO cells represent as a platform for testing therapeutics and targeting *TP53* associated oncogenes. Furthermore, platforms containing sgRNAs associated with TRE-Cas9 at the Col1a1 locus and rtTA at the Rosa26 locus were used to model the progression of Wilms’ tumor [33]. Chromosomal translocations in somatic cells, including duplication, inter-chromosomal translocations, insertions, deletions, and inversions, are considered as primary alterations of genetic material leading to cancer [34]. CRISPR/Cas9 system is also used to induce chromosomal abnormalities in vivo due to the possibility that the system offers to not only engineer short portions of DNA, but most importantly, large fragments in animal models [35].

All of these examples support the effectiveness of the CRISPR/Cas9 system in both disease modelling in animal and in vitro studies and its applicational potential in the field of gene therapy. While the use of this method is not yet widespread, the growing number of reported studies will most probably contribute to its better understanding necessary for its broad application in clinical medicine, as well as provide additional information about the genetic etiology of various diseases. Examples of ongoing clinical trials of immunotherapeutic agents that include a CRISPR/Cas9 element are presented in Table 1.

## 4. Precision Medicine and Immunotherapy in the Era of CRISPR/Cas9

Development of multiple drug resistance (MDR) in cancer prevents the insertion of therapeutic agents through the plasma membrane, limiting the effectiveness of classic cancer therapies [36]. Occurrence of such conditions often calls for introduction of more invasive treatment options, including radiation therapy, surgery, and targeted chemotherapy, depending on cancer development [37]. However, as most of the current therapeutic options are associated with significant risks and side effects, current research often focuses on decreasing the expression of soluble carrier proteins (SLC), passive transporters, ion-coupled transporters, and ion exchangers [38]. These types of studies stem from a fact that efflux transporters, including ABC transporters (ATP-binding-cassettes), significantly reduce the uptake of therapeutic agents by increasing ATP dependent efflux pumps that result in low intracellular drug concentration, thereby contributing to MDR [39].

Cancer originates as an accumulation of mutations, which brings the possibility of CRISPR/Cas9 use to target causative mutations in malignant cells [40]. CRISPR/Cas9, used as a diagnostic tool in treatment-resistant cancers, allowed to identify new therapeutic targets, including inhibitors of cancer multidrug resistance (MDR) [41]. Furthermore, genetic polymorphisms are also used as predictive factors for patients during treatment [42].

**Figure 3:**
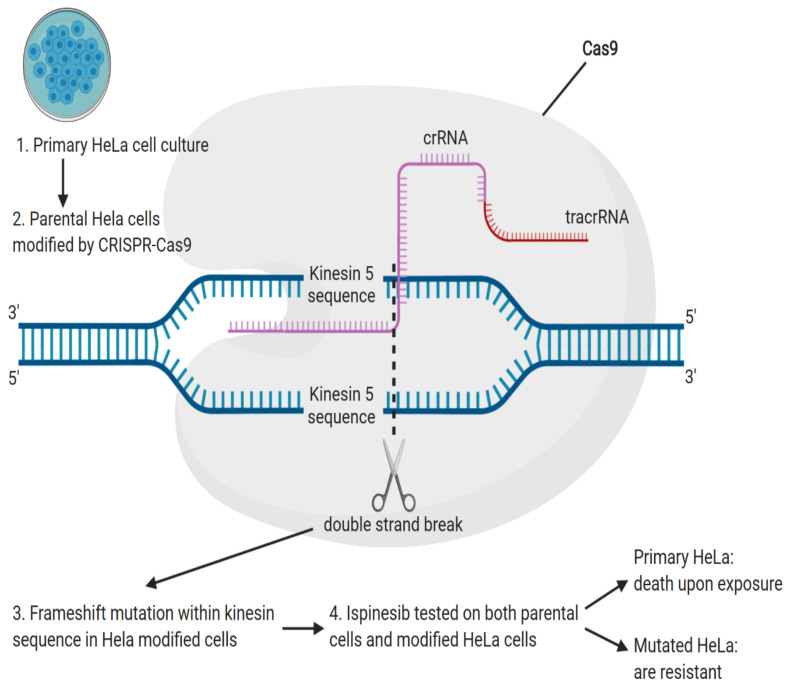
Following PCR confirmation of Kinesin 5 mutation in resistant cells, the primary culture of HeLa cell culture (parental) was plated (1). The parental cells were isolated and modified by CRISPR/Cas9 nickase (2) targeting the kinesin 5 sequence and yielding a frameshift mutation that resulted in HeLa clone daughter cells (3). Ispinesib treatment was performed on both parental and daughter cells (4). Parental cells died upon exposure, whereas daughter cells remained viable (5).

Chemotherapeutic drugs demonstrated to induce off-target mutations, with acquired drug resistance most commonly resulting from mutation substituting threonine with methionine at position 790 of the exon 20 (T790M) [43]. Recently, studies were conducted to analyze the resistance of cancer to ispinesib, a kinesin-5 inhibitor [44]. Isolated clones of cells resistant to ispinesib demonstrated a decrease in sensitivity by 70–300 fold [45]. In cells sensitive to ispinesib, monopolar mitotic spindle induced normal bipolar mitotic spindle formation, demonstrating that ispinesib resistance in mutated clones is independent of spindle assembly [46]. In a comparison of wild type parental cells with cells resistant to ispinesib, transcriptome sequencing and computational analysis allowed to identify C-terminal truncation mutations in Kinesin-5 clones [47]. For confirmation of ispinesib dependent resistance, CRISPR/Cas9 nickase was used to induce Kinesin-5 D130V and A133P mutation in HeLa cells (Figure 3). Effects of ispinesib were further tested on wild type HeLa cells that died upon exposure, with Cas9 modified HeLa cells exhibiting 150-fold resistance increase [48].

While the reports of CRISPR/Cas9 use in the context of precise medicine and immunotherapy are still sparse, the available results certainly make it a promising solution that could one day complement the approaches used to counteract such obstacles in cancer treatment as multiple drug resistance.

As cancer immunotherapy, specifically that targeting T-cells, is considered one of the greatest advances in recent biomedical research, gene therapy could improve diverse aspects of this technology. CARs (Chimeric Antigen Receptors), including the Novartis’s Kymriah, are an example of a promising advance, but so are the CRISPR/Cas9 based methods such as the knockout of the human leukocyte antigen, aiming to avoid immune rejection, as well as the knockout of PD-1 in T cells to avoid apoptosis and to stop cancer cells from escaping immunosurveillance [49,50].

## 5. Chimeric Antigen Receptors (CARs) for Cancer Immunotherapy

As malignant cells are programmed to tolerate self-antigens, they hold the ability to evade the host immune system through antigen alterations that enable them to stay undetected by lymphocytes [51]. Mutations in single or multiple genes encoding antigen processing factors can be targeted to develop clinical approaches for treatment of malignancies such as renal cell carcinoma, small cell lung carcinoma, and melanoma [52]. Chimeric antigen receptors (CARs) are synthetic immunoreceptors recently used cancer immunotherapy, serving as regulators of malignant cell adhesion to proteins presented on the extracellular membrane [53].

CAR T and CAR NK are the two most prominent CAR systems that modulate T lymphocytes and natural killer cells, respectively, offering a promising platform for anti-tumor immunotherapy. Using CRISPR-Cas9 screening, two research groups have lately discovered critical genes that prevent T lymphocyte and Natural killer cell mediated cytotoxicity. Leukemia screening allowed to sensitize the response of malignant cells to NK cells through targeting the modulating pathways their intracellular pathways, e.g., the interferon-γ signaling (IFN-γ) [54]. Another genome-wide CRISPR/Cas9 screen allowed to reveal genes responsible for resistance of human chronic-myelogenous-leukemia cells to NK cells induced by IFN-γ. This process was discovered to be governed by loss of gene encoding a ligand responsible for cytotoxicity, as well as by a gain of function mutation affecting antigen presentation that protects wild type cells [55]. Nonetheless, IFN-γ-mediated responses are of great concern, limiting ongoing research, due to IFN-γ having both tumorigenic and antitumorigenic characteristics [56]. The CAR T therapy, as for all the treatments personalized on a patient-by-patient basis, is highly expensive and time consuming, with solutions at the forefront of research concerning this clinical approach [57]. The presence of endogenous TCRs (T-cell receptors) in recipient cells may compete with the transgenic TCR for surface expression, with the possibility to use CRISPR/Cas9 to knock out the endogenous TCR-β, leading to improved cancer immunotherapies [58,59].

Even though CAR T cells caused significant improvement in therapies of hematological malignancies, there is still lack of clinical evidence concerning their benefits in solid tumors [60]. However, Rotolo et al. describe cytokine-induced natural killer cells and γδT as potential alternatives to chimeric antigen receptors in treatment of solid malignancies. Interestingly, genome-wide CRISPR/Cas9 library also enabled to identify loss of function of Apelin receptor, *APLNR*, regulating IFN-γ mediated responses to tumors, as responsible for impaired effector function of CD8^+^ T cells. The PD-1 blockade was a base for a range of checkpoint blocking strategies in cancer therapy, as application of the blocking antibodies, notably including disruption of the PD-1 gene by the Cas9 system was shown to exert significant anti-tumor effects [61]. Functional knockout of the PD-1 gene locus resulted in reduction of its expression and consequently upregulation of IFN-γ mediated responses, in turn leading to improved efficacy of human primary T cells in cancer patients [62]. Apart from previously mentioned limiting evidence concerning benefits of using CAR T cells in the treatment of solid tumors, recently conducted trials proved CRISPR/Cas9 ribonucleoprotein-mediated editing of the PD-1 gene in CAR T cells to result in relapse prevention and increased control of solid tumors, leading to CAR T cells being currently recognized to be as potent as the CAR NK systems [63].

Furthermore, targeted genome editing was recently used to identify genetic changes leading to acute lymphoblastic leukemia [64]. In vivo comparison at *TRAC* locus in an Acute Lymphoblastic Leukemia (ALL) mouse model performed between cells with retroviral vector chimeric antigen receptors (RV-CARs), *TRAC*-CARs cells, and TCR-disrupted cells expressing chimeric antigen receptor [(RV-CAR(+)-TCR(-))], demonstrated greater response and prolonged survival of *TRAC*-CARs induced cells [65]. While some limitations of the chimeric antigen receptor were further identified following bone marrow transplant of CAR T cells, inhibition of metastasis was observed in *TRAC*-CAR despite continuous expression of CD19 [66]. In addition to anti-cancer efficacy, CARs T-cells differ in terms of T-cell differentiation and expression of terminal effector cells [(CD45(+), CD62L(−)] [67]. In total, 50% of conventional CAR T cells demonstrate differentiation into terminal effector cells and expression of exhaustion markers when compared to TRAC-CARs [68]. The latter expressed less than 2% exhaustion and retention markers of the effector cells [65]. The use of animal models in the context of acute lymphoblastic leukemia also delivered better results for the treatment of B-Cell ALL with CARs [69].

**Figure 4:**
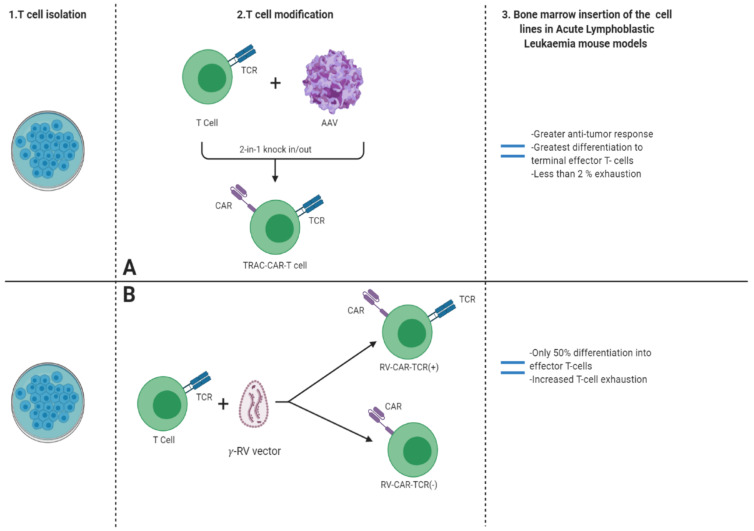
Firstly, T cell isolation was conducted (1). Then, in a comparative study, some T-cells were modified using an adeno-associated virus, which exhibited a gRNA that targets TCR (T-cell receptor) and a sequence for CAR (Chimeric Antigen Receptor) expression. This process resulted in formation of CAR-T-cells that lacked TCR (2A). Other cells were modified using a γ-retroviral vector, yielding CAR-T-cells both positive and negative for TCR (2B). All three cell lines were then inserted in the bone marrow of an acute lymphoblastic leukemia mouse model (3). Comparative effectiveness was assessed, proving the CAR-T-cells modified by CRISPR/Cas9 to be superior in the form of treatment.

Furthermore, clinical trials involving CAR T cells have shown positive results involving 19 patients with neuroblastoma, treated with CAR-expressing cytotoxic T-lymphocytes and CAR-expressing adoptive T-cells [70]. In another clinical trial, 19 patients suffering from sarcoma were treated with CAR T cells sensitized for human epidermal factor 2 (HER2) with survival rate post-treatment extended from 5.1 to 29.1 months [71]. As previously mentioned, although immunotherapy delivered positive results in acute lymphoblastic leukemia, neuroblastoma, and hematologic disorders, its efficacy in clinical studies concerning engineered T cell therapies for solid tumors demonstrated different responses [72]. Unlike hematologic malignancies, solid tumors maintain a barrier-forming toxic tumor microenvironment [73]. Additionally, the presence of heterogeneity of cell surface antigens, even between cancer cells within solid tumors, renders CAR T cells targeting inefficient. In a glioblastoma study, first generation CD3 positive CAR T cells were used to specifically target the epidermal growth factor receptor variant III (EGFRvIII) expressed in glioblastoma cells [74]. The results demonstrated decreased expression of EGFRvIII, followed by an increase in programmed cell death factors, specifically PD1 immune suppressants. In a subsequent study, second-generation CAR T cells were genetically engineered using the CRISPR/Cas9 tool and an adeno-associated viral vector containing the CAR sequence to achieve specific CAR integration, in contrast to first-generation CAR T cells (Figure 4) [75]. CRISPR/Cas9 was also used to perform functional knockouts of β-2 micro-globulin and *TRAC* loci. The effect was the greatest in mice given engineered CAR-Tcell-EGFRvIII-PD-1, resulting in significant prolongation of their life [76]. There are also both ongoing and completed clinical establishing the efficacy of knockouts through intra-ventricular infusion in animal models. One of the recently completed clinical trials involved genetic targeting of patients suffering from gastric cancer. The study included the collection of peripheral blood lymphocytes prior to PD-1 knock out. The efficiency of PD-1 knock out was further tested in vivo following the reinjection, showing promising results [77]. Finally, another trial involved patients suffering from relapsed multiple myeloma, synovial sarcoma, and myxoid cell liposarcoma, characterized by low expression of NY-ESO-1 protein prognosis factor. Patients’ T-cells were transduced with lentiviral vectors expressing NY-ESO-1, followed by functional knock out of both TCR and PD-1 [75].

All of these results indicate CAR-Ts as a potentially effective vector of anti-cancer treatment, with a growing amount of research focused on its application (Table 2). Further use of the CRISPR/Cas9 system has a considerable potential in studies attempting to overcome some of the limitations associated with this approach, as well as improve its efficacy and specificity.

## 6. Limitations of the CRISPR/Cas9 System

CRISPR/Cas9 editing efficiency is dependent on the specificity of the 20 nucleotide-long stretch within guide RNA complementary to the target sequence [85]. The most concerning limitation of the system is the off-target effects resulting in nonspecific cuts, further leading to unwanted mutations [86]. Specifically, when targeting cancerous changes, nonspecific cuts may have a dominant-negative effect on the function of oncogenes and tumor suppressor genes, leading to either gain of function or loss of function mutations [87]. To alleviate these kinds of negative side effects, some researchers suggested modifying the length of gRNA aiming to decrease nonspecific INDELs, unwanted insertions, or deletion in the targeted genome [88]. In a recently completed clinical trial, the length of the gRNA was increased through addition of two guanines to its 5′ end, which resulted in decreased frequency of off-target modifications [89]. Nonetheless, the modification caused an unwanted decrease in the efficiency of on-target effects. In another trial, shortened gRNAs demonstrated to hold better on-target effects than their original length counterparts, but were still accompanied by the presence of off-site effects [90]. In an alternative approach, researchers modified nicking enzymes by mutating RuC and HNH catalytic residues of Cas9. Nonetheless, the replacement of Cas9 nuclease activity by nickase activity was not efficient. However, a double nicking strategy was proven more efficient in inducing HDR and NHEJ [91]. Another trial demonstrated a correlation between the amount of Cas9 protein and its ability to induce off-target mutations. Decreasing the activity of Cas9 improved editing specificity. However, the mentioned improvements still result in either decreased editing activity or increased off-target mutations [92]. The frequency of off-target mutations may increase in the presence of single nucleotide mutations (SNP), causing errors in the analysis of cell lines used to validate designed gRNA or targeting molecules and leading to unprecise gene modifications [93]. Furthermore, guidelines for defining off-target mutations in DNA are still some way from reaching a consensus [94]. Additional limitations of the CRISPR/Cas9 system include the size of the catalytic window, which is around 4–5 nucleotides, causing low specificity and efficiency, as well as the requirement of a PAM sequence and the high specificity of the target base [95,96]. Furthermore, the mosaicism happening when the cells divide during genome editing is also a potential limitation of this technology, as daughter cells may not carry the CRISPR induced modifications intact or undergo incomplete targeting [97,98]. Another study determined molecular interactions and crystal structure of Cas9-gRNAs. This investigation of the molecular interactions between gRNAs and target DNAs allowed to create new designs, including *Streptococcus pyogenes* Cas9, SpCas9, and *Staphylococcus aureus* Cas9, SaCas9, aiming to improve the specificity of binding [99]. In a comparative study, efficiency of saCas9 was established, and specificity of saCas9 was tested against spCas9. In both cases, cleaved targets of a comparable rate were recorded in association with high specificity, small size, and, most importantly, the ability of saCas9 and spCas9 to work with various gRNA lengths [100]. To overcome the inhibitory effect of PAM sequences on the action of Cas9, new bacterial CRISPR-Cpf1 crystal structures were analyzed to establish new variants, assembled to target different PAM sequences [101]. Furthermore, to delivery of Cas9 protein in the form of ribonuceloprotein (RNP) both in vitro and in vivo was also shown to pose a significant challenge. Nonetheless, a supramolecular polymer system comprising of β-cyclodextrin-conjugated low molecular weight polyethyleneimime and disulfide-bridged biguanidyl adamantine was recently employed to overcome this limitation. Addition of the polymer aided transformation of colorectal cancer cells and 293T cell lines through the interaction with Cas9 through strong hydrogen bonding and multiple salt bridges [102]. Moreover, the limitations surrounding gene therapy span from ethical terms to scientific uncertainties, and its application in clinical studies and human testing is still a matter of discussion. Assessing the risk is one of the ethical challenges that needs to be faced, as even though preclinical animal and cell cultures research rarely shows risk, some clinical trials have adverse side effects, including the death of the subjects [103].

All of the above research shows that while the use of the CRISPR/Cas9 method is still characterized by significant limitations, there is a growing amount of studies aiming to find solutions for the different types of obstacles preventing its widespread use.

## 7. Conclusions

Cancer remains the second cause of death worldwide, despite a significant number of therapeutic advancements. CRISPR/Cas9 is holding great promise in targeting drug-resistant cancers and correcting tumor causative mutations in the human genome. Not only CRISPR is favored over other genome editing tools due to its price but, most importantly, specificity and simplicity in designing gRNA to match specific targets. Furthermore, as immunotherapy is becoming more effective in a multitude of cancers, due to its association with the genome editing system, many ongoing clinical trials explore CRISPR/Cas9 in the context of cancer-resistant immuno-therapeutic agents to aid the immune system in combating malignancies. CRISPR systems are also used in animal models to identify novel mutations and candidate genes. Incorporating CRISPR into cancer treatment and research shows great promise, as demonstrated in vitro and in vivo. However, several limitations still need to be addressed in the future to establish the true potential of these methods and to enable the possibility of its widespread use, such as off-target site mutations, resulting in nonspecific restrictions of DNA upstream of the Protospacer Adjacent Motifs (PAM), the lack of ethical agreements, and no scientific consensus concerning risk evaluation. Hence, further research is needed to fully elucidate the risks and benefits of gene therapy, to ensure that both the scientific community and public opinion will allow for its worldwide application in clinical settings.

## Figures and Tables

**Table 1:** The ongoing clinical trials of immunotherapeutic agents that include a CRISPR/Cas9 element, as found in clinicaltrials.gov.

Disease	Country	Phase	Cell Type	Target	Intervention	ID
Gastrointestinal Epithelial Cancer	USA	I/II	Tumor Infiltrating Lymphocytes (TIL)	CISH (Cytokine-induced SH2 protein)	Drug: Cyclophosphamide Drug: Fludarabine Biological: Tumor-Infiltrating Lymphocytes (TIL) Drug: Aldesleukin	NCT04426669
Gastrointestinal Neoplasms						
Gastrointestinal Cancer						
Colorectal Cancer						
Pancreatic Cancer						
Gall Bladder Cancer						
Colon Cancer						
Esophageal Cancer						
Stomach Cancer						
B Cell Leukemia	China	I/II	B-cells	CD19, CD20, or CD22 Knockout	Biological: Universal Dual Specificity CD19 and CD20 or CD22 CAR-T Cells	NCT03398967
B Cell Lymphoma						
B Cell Leukemia	China	I/II	B-cell	CD19	Biological: UCART019	NCT03166878
B Cell Lymphoma						
Refractory B-cell malignancies	USA	I/II	bVCB-cel B-cell	Creation of a CD19-directed T cell	CD19-directed T-cell immunotherapy	NCT04035434

**Table 2:** Effects of cancer-specific induced mutations on metastasis and malignancy of different cancers, including examples of the benefits of CRISPR/Cas9 in the discovery of new therapeutic targets for more effective cancer treatment.

Cancer Type	CRISPR/Cas9 Modifications	Therapeutic Contributions	Reference
Pancreatic cancer	KDM6A knockout	Increase in the aggressiveness of pancreatic ductal adenocarcinoma	[78]
Breast cancer	MIEN-1 knockout	Increase of progression and metastatic potential	[79]
SCLC	P107 knockout	Inhibition of tumor suppressor activity	[80]
Prostate cancer	GPRC6A knockout	Inhibition of cell proliferation	[81]
Endometrial cancer	MUC1 knockout	Inhibition of EGFR expression	[82]
Breast cancer	miR-644a knockout	Inhibition of growth, metastasis, and treatment resistance	[83]
Prostate cancer	NANOG and NANOGP8 knockout	The decrease in malignant potential	[84]
